# Synthetic Virus-Like Particles Target Dendritic Cell Lipid Rafts for Rapid Endocytosis Primarily but Not Exclusively by Macropinocytosis

**DOI:** 10.1371/journal.pone.0043248

**Published:** 2012-08-14

**Authors:** Rajni Sharma, Arin Ghasparian, John A. Robinson, Kenneth C. McCullough

**Affiliations:** 1 Institute of Virology and Immunoprophylaxis, Mittelhäusern, Switzerland; 2 Department of Chemistry, University of Zürich, Zürich, Switzerland; Imperial College London, United Kingdom

## Abstract

DC employ several endocytic routes for processing antigens, driving forward adaptive immunity. Recent advances in synthetic biology have created small (20–30 nm) virus-like particles based on lipopeptides containing a virus-derived coiled coil sequence coupled to synthetic B- and T-cell epitope mimetics. These self-assembling SVLP efficiently induce adaptive immunity without requirement for adjuvant. We hypothesized that the characteristics of DC interaction with SVLP would elaborate on the roles of cell membrane and intracellular compartments in the handling of a virus-like entity known for its efficacy as a vaccine. DC rapidly bind SVLP within min, co-localised with CTB and CD9, but not caveolin-1. In contrast, internalisation is a relatively slow process, delivering SVLP into the cell periphery where they are maintained for a number of hrs in association with microtubules. Although there is early association with clathrin, this is no longer seen after 10 min. Association with EEA-1^+^ early endosomes is also early, but proteolytic processing appears slow, the SVLP-vesicles remaining peripheral. Association with transferrin occurs rarely, and only in the periphery, possibly signifying translocation of some SVLP for delivery to B-lymphocytes. Most SVLP co-localise with high molecular weight dextran. Uptake of both is impaired with mature DC, but there remains a residual uptake of SVLP. These results imply that DC use multiple endocytic routes for SVLP uptake, dominated by caveolin-independent, lipid raft-mediated macropinocytosis. With most SVLP-containing vesicles being retained in the periphery, not always interacting with early endosomes, this relates to slow proteolytic degradation and antigen retention by DC. The present characterization allows for a definition of how DC handle virus-like particles showing efficacious immunogenicity, elements valuable for novel vaccine design in the future.

## Introduction

Dendritic cells (DC) [1] play a crucial role initiating and promoting immune response, and are essential for the development of robust, efficacious immune defences. An important characteristic is the capacity of DC to employ different endocytic pathways towards efficient antigen internalisation and processing; reviewed by Lin and co-workers [2]. DC possess the potential for selective application of endocytic pathways dependent or independent of clathrin, caveolae, lipid raft mobilisation and/or macropinocytosis; antigen delivery routes can employ endosomal pathways and/or the endoplasmic reticulum [3], [4], [5], [6], [7], [8]. This diversity of processing routes provides DC with a high capacity for promoting antigen delivery and processing. Yet, these same endocytic pathways are involved in degrading internalised material. It has been reported that DC display a more limited protease activity compared with macrophages; this was related to a slower *in vivo* degradation of internalized antigens, and antigen retention for extended periods, which favoured antigen presentation [9].

**Figure 1:**
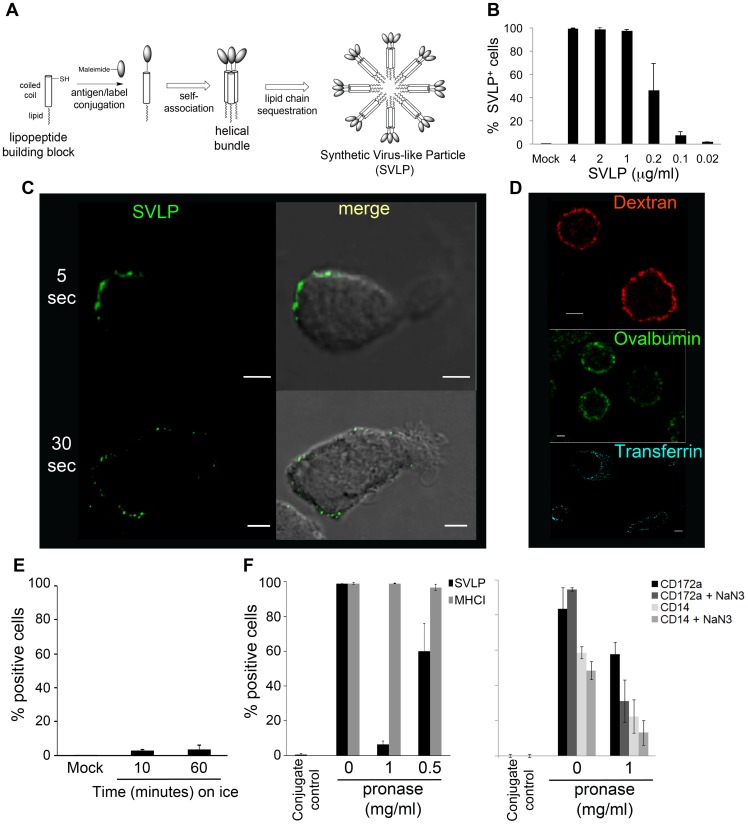
SVLP binding to DC. (A) Graphic representation of SVLP. (B) DC were incubated with different concentrations of SVLP in medium without serum on ice for 20 min. Samples were washed and analysed by flow cytometry. (C) Pre-chilled DC were incubated with 2.5 µg/ml for SVLP for 5 or 30 seconds, then washed and fixed (10min, room temperature, 4% w/v p-formaldehyde) prior to analysing by confocal microscopy. Scale bars: 5 µm. (D) DC were incubated with high molecular weight dextran-546 (500 kDa), Ovalbumin-488, transferrin-546 (artificially coloured blue to aid visualisation) or PBS (mock) for 1 min at 39°C followed by analysis using confocal microscopy. Scale bars: 5 µm. (E) Pre-chilled DC were incubated with Ovalbumin-488 or PBS for 10 or 60 min on ice, followed by washing and analysis by flow cytometry. (F) Receptor-mediated binding of SVLP. DC were treated with pronase for 25 min at 39°C. Cells were then pre-cooled on ice for 30 min and washed 5 times. SVLP (1 µg/ml), or antibody against MHCI, CD172a or CD14, were added for 20 min on ice (antibody binding was then detected with Alexa_488_-conjugated anti mouse immunoglobulin F(ab')_2_). The cells were also treated with 3 mM NaN_3_ to impair recycling of CD172a or CD14. Cells were analysed by flow cytometry. For the % SVLP positive cells, difference was significant between cells treated with pronase at “0.5 mg/ml” and “1 mg/ml” (p = 0.004), and also between “no pronase” and “0.5 mg/ml pronase” (p = 0.014). Results (B, E–F) are means of three samples ± s.d.

DC have been labelled professional antigen presenting cells [10]. Immature DCs display more efficient endocytic processes than mature DCs [11]. While appropriate targeting of vaccines to DC would favour immune defence development, this area still requires clarification concerning the characteristics of DC endocytic uptake. With particulate delivery vehicles [12], [13] being promoted as vaccine carriers targeting DC for efficient immune defence induction [14], [15], [16] defining endocytic uptake is important for vaccine design. Although there is information on model proteins such as ovalbumin, there is less information on how DC handle more complex vaccines. Accordingly, the aim of the present work was to characterize how DC handled Synthetic Virus-Like Particles (SVLP). These are complex antigenic structures with an already proven capacity for efficient induction of IgG responses; importantly, this is achieved without adjuvant requirement in rabbits [17] and pigs (R.S, unpublished data). SVLP are formed by lipopeptide self-assembly into spherical nanostructures in the 20–30 nm size range, which resemble small virus-like particles in their shape, size and chemical composition [18], [19]. Like naturally-derived VLPs, SVLP can be exploited for multivalent presentation of antigens across their surface (Fig. 1A–S1A) [17] and several SVLP constructs have previously been shown to induce strong immune responses in experimental systems without co-administration of adjuvants [18], [20].

Despite the work showing efficient immune response induction, there is no knowledge on SVLP interaction with DC. We hypothesised that their interaction with DC could elaborate on the characteristics of DC-antigen interactions relating to efficacious induction of immune responses. Moreover, the SVLP should provide the means of defining how DC handle a complex virus-like structure. The efficiency of the SVLP vaccine allows these characteristics to be defined without the encumbrance of overlaying adjuvant activity. Accordingly, the present work characterised the manner by which DC bind and internalise SVLP, relating to the involvement of different intracellular organelles and compartments known to be important for the variety of DC functions.

## Results

### SVLP interact with DC in a time and concentration dependent manner

The initial analyses sought to determine the kinetics of SVLP interaction with DC, which was performed over a 20 min period on ice to impede the internalisation processes. Flow cytometry analyses showed SVLP binding in a concentration dependent manner; optimum binding with 1 µg/ml resulting in 98% SVLP-positive cells (Fig. 1B). This was confirmed by confocal microscopy, with which the strongest signal was obtained using 2.5 µg/ml SVLP (data not shown). The binding to DC on ice was also time-dependent and rapid, being observed as early as the 5-second time point (plus the time for washing prior to fixation of the DC with 4% w/v p-formaldehyde to prevent further activity) (Fig. 1C). Binding appeared to peak at 5 min (data not shown).

Such a rapid interaction of SVLP with DC was not unique. High molecular weight dextran, ovalbumin and transferrin also interacted rapidly (Fig. 1D), but contrasted with SVLP in requiring metabolically active cells; only a low signal was obtained for binding on ice (Fig. 1E shows ovalbumin as an example). Although SVLP binding to DC on ice was variable with time, 5 independent experiments showed that the interaction depicted in Fig. 1C was consistently rapid.

This rapid SVLP binding to DC may have reflected an ionic or a receptor-mediated interaction. Consequently, pronase (titrated for its optimum concentration and incubation time; data not shown) was used to pre-treat DC at 39°C. The cells were then washed and cooled on ice prior to adding SVLP or antibody against MHC Class I (MHCI), CD172a or CD14 on ice (Fig. 1F). Labelling of the cell surface markers was to confirm the proteolytic effect of pronase on sensitive cell surface protein receptors (CD172a, CD14), but not all molecules (MHCI); the pronase sensitivity of CD172a and CD14 was enhanced by treating the cells with 3mM NaN_3_ to impair receptor recycling (Fig. 1F). A 90% reduction in binding was observed with SVLP, compared to cells not treated with pronase (Fig. 1F). This was considered to be indicating the removal of a putative protein receptor for SVLP binding.

### SVLP internalisation by DC

**Figure 2:**
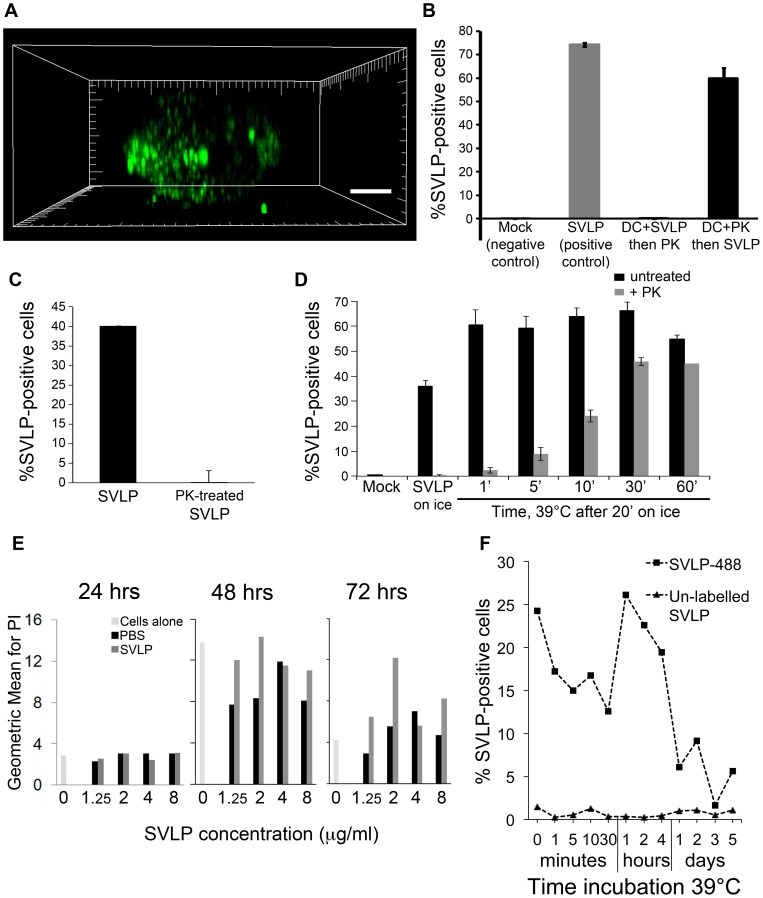
Internalisation of SVLP by DC. (A) DC incubated with 2.5 µg/ml SVLP (green) for 30 min at 39°C. High resolution stacks (1024×1024 voxels) were prepared, and 3D-imaging preformed using IMARIS. Scale bar: 5 µm. (B) SVLP (1 µg/ml) were added to pre-chilled DC (SVLP) or not (Mock) for 30 min on ice; pre-chilled DC were treated with PK before (DC + PK then SVLP) or after (DC + SVLP then PK) incubation with SVLP on ice. PK was added for 30 min on ice, then 10% serum added to “neutralise” PK activity. Cells were analysed by flow cytometry. The % Alexa_488_ positive cells were significantly different between SVLP (cells not treated with PK; “SVLP (positive control)”) and “DC + PK then SVLP” (p = 0.006). (C) SVLP (1 µg/ml) were first pre-treated with PK as in (B). The treated SVLP were added to DC on ice for 30 min. Cells were washed and analysed by flow cytometry. (D) Pre-chilled DC were pulsed with 1 µg/ml SVLP for 20 min on ice and then shifted to 39°C for different time points, followed by treating or not with PK for 30 min on ice, and analysed by flow cytometry. (E) DC were incubated with different concentrations of SVLP for 24, 48 or 72 hrs at 39°C. Following washing, the cells were treated with PI and analysed by flow cytometry. The “0” on the x-axis indicates the cell control untreated with SVLP. (F) DC were incubated with 1 µg/ml SVLP for 20 min on ice, then different incubation time at 39°C, followed by flow cytometry analysis. Results are means of three samples ± s.d.

The capacity of DC to internalise bound SVLP employed incubations at 39°C (the resting body temperature of the porcine donors of the DC). Following incubation for 30 min at 39°C, confocal microscopy showed SVLP internalisation by DC using 3–D imaging (Fig. 2A).

**Figure 3:**
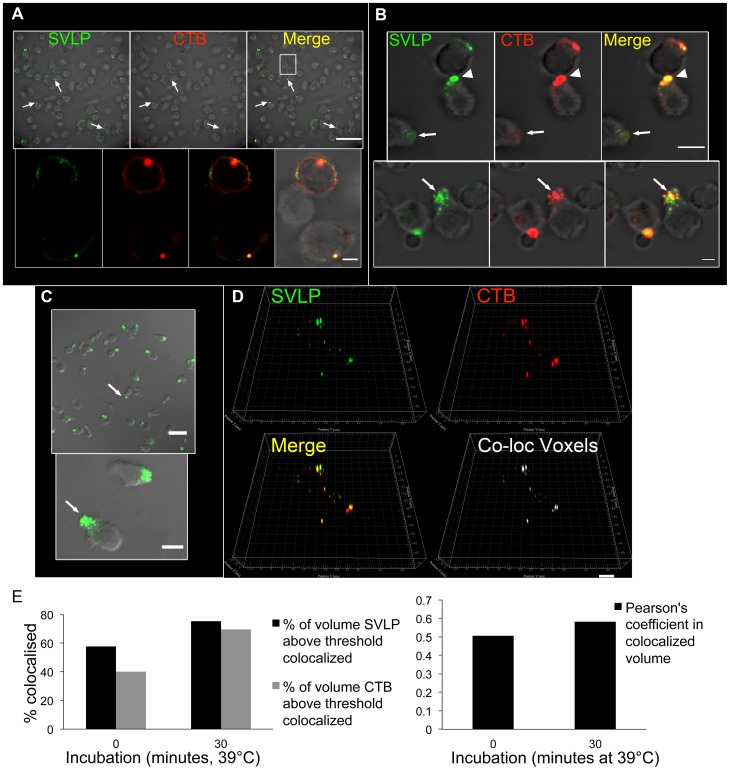
SVLP association with lipid rafts. (A) Pre-chilled DC were treated with 2.5 µg/ml SVLP (green) and 5 µg/ml CTB (red), followed by 30 min incubation on ice and washing. Samples were fixed and analysed by confocal microscopy. Arrows indicate SVLP and CTB localisation. The white square is the zoomed area in the lower panel; the lower panel shows a different section to that in the upper panel, to demonstrate that areas with apparently no co-localisation of the SVLP with CTB could overlay areas showing co-localisation. Scale bars: 50 µm (upper panel) and 5 µm (lower panel). (B) DC treated as (A) were given warm medium and shifted to 39°C for 10 min. Arrowheads indicate SVLP and CTB co-localisation on polarised membranes at intercellular contacts; arrows indicate internalisation at polarisation sites. Scale bars: 10 µm (upper panel) and 5 µm (lower panel). (C) Pre-chilled DC incubated with 2.5 µg/ml SVLP (green) on ice for 20 min followed by washing and shifting to 39°C for 10 min. The image in the lower panel is a zoom of the white square in the upper panel. Arrows show SVLP associated with lamellipodia. Scale bars: 30 µm (left) and 10 µm (right). (D) DC were pulsed with 2.5 µg/ml SVLP and 5 µg/ml CTB for 30 min on ice, then shifted to 39°C for 30 min. High-resolution stacks, 3D images and co-localisation analysis, using IMARIS, demonstrate co-localisation of SVLP with CTB; this included algorithmic analysis to identify co-localised voxels. Scale bar: 8 µm. (E) Algorithmic analysis of the results from (D) performed using IMARIS.

In order to confirm that the SVLP were internalised, PK treatment was employed to remove material from the cell surface. SVLP were interacted with DC for 30 min on ice, followed by PK treatment for a further 30 min on ice. This resulted in an abrogation of the SVLP fluorescent signal (Fig. 2B; “DC + SVLP then PK” compared with “SVLP(positive control)”). If the DC were pre-treated with the PK for 30 min on ice prior to addition of the SVLP (Fig. 3B; “DC + PK then SVLP”), there was only a minor reduction in the signal obtained. It was considered that these results demonstrated a sensitivity of the SVLP to digestion by PK, whereas the receptor(s) for SVLP binding was apparently insensitive. This sensitivity of the SVLP was confirmed by pre-treating with PK on ice prior to incubation with DC; again the fluorescence signal was lost with the treated SVLP (Fig. 2C). Subsequent experiments in which SVLP internalisation required confirmation used this sensitivity of the SVLP to the PK treatment on ice – resistance to PK was taken as indicative of internalisation.

The rate of SVLP internalisation by DC was analysed by pulsing the cells with SVLP on ice for 20 min, washing to remove any unbound particles, then shifting to 39°C. After different times at 39°C, treatment with PK on ice for 30 min allowed definition of the degree of internalisation (PK activity was neutralised by addition of 10% porcine serum). Internalised SVLP, and therefore protected from the PK treatment, were observed as early as one min after shifting to 39°C (Fig. 2D). However, only a minor proportion of the cells showed evidence of internalised material at this time. There was a gradual increase with time for the number of cells displaying internalised material, reaching maximum uptake at 30 min (Fig. 2D).

### SVLP are non-toxic for DC

Despite the apparently efficient internalisation of SVLP by DC, this did not exclude an eventual toxicity for the cells. Accordingly, DC were given 1.25, 2, 4, and 8 µg/ml of SVLP, and incubated for 24, 48 and 72 hrs at 39°C without removal of the SVLP. After harvesting at these different time points, the cells were stained with propidium iodide (PI) to quantify dead or damaged cells. No cell death was observed above that obtained with the “cell only” control, when analysed at 24 and 48 hrs (Fig. 2E). A slight increase in the number of PI-positive cells was seen with the SVLP after 72 hrs, but this was not related to the SVLP concentration, and with most concentrations represented <10% cell population. Statistical analysis showed that the numbers of PI^+^ cells with SVLP-treated cells were not significantly different from “PBS” or “cells alone”. It was therefore considered that SVLP are non-toxic for DC.

### Kinetics of SVLP association with DC

While the above results focussed on the kinetics of uptake and internalisation, it was also important to determine if the SVLP were degraded over time. Following SVLP pulsing of DC on ice, and washing prior to the 39°C shift, it was noted that between 4 and 24 hrs the fluorescent signal declined rapidly, reaching a minimum close to the background signal by a 72 hrs (Fig. 2F). Although a few positive cells could be found at later times, this was rare and inconsistent.

### SVLP association with lipid rafts

The association of SVLP with particular subcellular fractions gave no information on the manner of uptake; DC operate various endocytic pathways, which can determine the fate of the internalised material. An early characteristic defining particular endocytic pathways is the involvement of lipid raft mobilisation, identifiable by CTB binding to five molecules of GM1 [21]. Within 30 min of incubation with DC on ice, CTB (under predetermined optimum conditions for visualising lipid rafts) showed an irregular pattern of interaction (in patches) with the plasma membrane (Fig. 3A, “CTB”); This pattern can be seen more distinctly in the zoomed images of the lower panel for Fig. 3A. This was considered to reflect the irregular distribution of lipid rafts throughout the membrane. These pre-chilled DC were also given SVLP together with CTB for 30 min on ice, washed to remove unbound particles and CTB, and shifted to 39°C for different periods of time. At 0 min (the time of temperature shifting), the CTB and SVLP were seen together, distributed about the cell membrane (Fig. 3A). Despite a clear association between the CTB and SVLP signals, there were also sites of SVLP interaction without any detectable CTB. The lower panels of Fig. 3A are zoomed on to the white square shown in the upper panel “Merge”. Therein, association of the SVLP and CTB signals can be seen, as well as areas of red SVLP devoid of a green CTB signal. Moreover, the lower panel shows a different z-section to that in the upper panel, to demonstrate that sections showing apparently no associating (as in the white square of the upper panel) can display association in a different z-section (lower panels). Such observations that not all SVLP binding can be observed associated with sites of CTB binding may reflect additional SVLP interaction with the cell membrane at sites devoid of GM1, or a disparate distribution of the CTB to the GM1 sites.

Within 10 min at 39°C, clear polarisation of the CTB together with the SVLP was observed (Fig. 3B), reminiscent of accumulation at the leading edge of the cell. There were also indications that CTB plus SVLP accumulation could occur at sites of intercellular contact (Fig. 3B, arrow head in upper panel). An apparently parallel internalisation of CTB with SVLP was visible at polarisation sites (Fig. 3B, arrow in lower panel). This accrual of SVLP at polarised sites related to the observations showing SVLP accumulating at sites of lamellipodia and filopodia formation (Fig. 3C, arrows). The formation of this leading edge was independent of SVLP binding, being observed in control DC cultures upon shifting from ice to 39°C (data not shown).

At the time of observed maximum internalisation (maximum PK-resistance; see Fig. 2D), the SVLP remained associated with CTB (Fig. 3D). Although there were SVLP-containing structures in which CTB was absent, co-localisation algorithm analysis confirmed a high degree of co-localisation between the two (Fig. 3D, E). Such frequent association of SVLP with CTB indicated lipid raft involvement in DC uptake of SVLP. An important lipid raft-dependent endocytic route involves caveolar uptake. However, this appeared not to be involved, because no co-localisation with caveolin-1 was detectable (data not shown).

### Influence of cholesterol depletion on SVLP binding to DC

**Figure 4:**
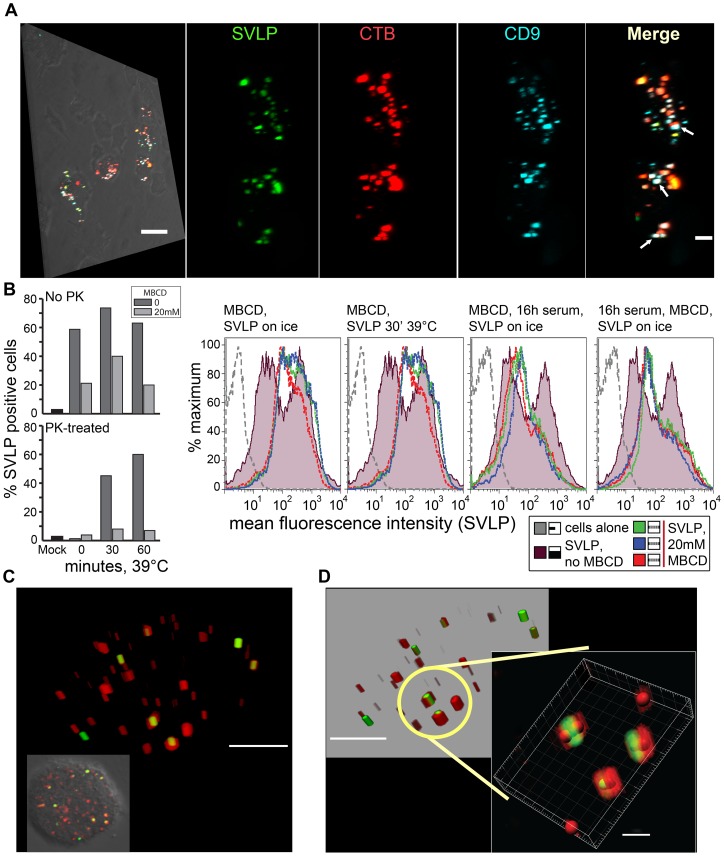
SVLP associating with early endosomes and CD9. (A) Pre-chilled DC were incubated with SVLP and CTB as in Fig. 3, or with antibody against CD-9 for 30 min on ice. Samples were then washed and shifted to 39°C for 2 hrs, followed by confocal microscopy analysis. High-resolution stacks prepared using IMARIS are shown for each label, arrows indicating co-localisation. Scale bars: 20 µm (left image), 5 µm (zooms). (B) DC were treated with 20 mM MBCD for 20 min at 39°C, followed by washing to remove the MBCD and pre-chilling for 30 min on ice. DC were then pulsed with 2.5 µg/ml SVLP for 20 min on ice (“0” time point) and then washed with pre-chilled medium, prior to receiving fresh warm (39°C) medium containing 2 mM MBCD and shifting back to 39°C. DC were treated or not with PK to define internalisation at the time points shown. Analyses were by flow cytometry; all differences at the 39°C time points between 0 MBCD and 20 mM MBCD treatment are significant, as are the differences between “No PK” and “PK-treated” for the 20 mM MBCD samples (p≤0.001). (C) DC were incubated with 2.5 µg/ml SVLP at 39°C for 10 min, then fixed/permeabilised and co-labelled with anti-EEA-1 antibody prior to confocal microscopy analysis. Scale bar: 3 µm. (D) A 3-dimensional analysis of the circle shown in (C); the right image shows a further analysis of the circle in the left image, analysed with the aid of Spots module in IMARIS to define centre points for SVLP (green) and EEA-1 (red) labelling. Scale bars: 3 µm (blend), 1 µm (zoom; spot module to enhance visualisation, filtered on mean intensity).

SVLP association with lipid rafts was confirmed using antibody against CD9, due to the report of CD9 in lipid rafts of human monocytes [22]. DC were incubated as before with SVLP and CTB for 30 min on ice, but now also with antibody against CD9. The cells were then shifted to 39°C for 2 hrs. SVLP were observed associated with both CD9 and CTB (Fig. 4A, arrows). These results confirmed that SVLP primarily associated with lipid rafts; although not all SVLP were visualised together with CTB, they did associate with at least CTB or CD9.

Considering that lipid rafts are cholesterol-rich membrane regions, the importance of cholesterol in SVLP uptake employed MBCD to deplete cholesterol. DC were treated with MBCD for 20 min at 39°C, followed by washing to remove MBCD and chilling on ice. SVLP binding (20 min on ice, in the absence of any further addition of MBCD) was clearly impaired (Fig. 4B, “No PK”, “0” time point); statistical analysis showed the difference between untreated and MBDC-treated cells to be p<0.001. As expected, any SVLP still binding was on the cell surface, confirmed by its sensitivity to PK-treatment (Fig. 4B “PK-treated”). Fig. 4B shows the effect of 20 mM MBCD; similar results were obtained with 10mM and 40mM MBCD (data not shown). The cultures were again washed to remove unbound SVLP, and fed with pre-warmed medium (39°C) containing 2 mM MBCD to maintain the cholesterol depletion, and shifted back to 39°C. The gradual uptake of SVLP with time (time-dependent increase in PK-resistance at 39°C) was also significantly (p<0.001) impaired (Fig. 4B, “No PK” compared with “PK-treated).

MBCD-treated and untreated cells, followed by addition of SVLP for 20 min on ice, were overlayed for analysis. Thereby, the reduction in the SVLP signals was clearly observable (Fig. S2A). Interestingly, the SVLP signal was not abrogated following MBCD treatment (also seen in Fig. 1B “No PK”, “0”). Accordingly, the analyses were repeated, but using CTB in place of SVLP. In this case, there was no apparent effect of the MBCD treatment (Fig. S2B). The MBCD pre-treatment followed by SVLP addition on ice was also repeated, but with a 10-fold higher concentration of SVLP (10 µg compared with 1 µg). With this 1 µg sample, the SVLP signal was somewhat weaker (Fig. S2C, left panel, compared with Fig. S2A, upper left panel). Nevertheless, the inhibitory effect of MBCD pre-treatment was still observable (Fig. S2C, second and third panels). The signal obtained with the 10 µg SVLP was much stronger (Fig. S2C, right panel, grey/black unshaded histograms), but now the MBCD did not reduce had little influence on the signal obtained. This higher concentration of SVLP gave a double peak of fluorescence intensity, whereas the MBCD showed inly a single peak, but of the triplicate samples there was no significant reduction in the SVLP signal.

We did not attempt to increase the MBCD concentration, due to the increase toxicity for the cells. Indeed, the 20mM MBCD treatment led to increased cell membrane permeability (Fig. S2D middle panel), which could not be restored (Fig. S2D right panel). For these reasons, we can only interpret the effect of the MBCD on SVLP binding. Overall, it is difficult to interpret the influence of MBCD beyond the removal of cholesterol. Indeed, the major source of information on SVLP interaction with DC is provided by the colocalisation studies of SVLP with CTB and CD9 (Fig. 4A).

### Fate of SVLP in DC and involvement of intracellular organelles and compartments

The internalisation of SVLP into cytoplasmic and membrane fractions raised the question of which endocytic processes and intracellular compartments were involved, and in particular if more than one pathway was operative. With primary DC and monocyte-derived DC, DNA plasmid transfection is highly inefficient [23], rendering analyses of intracellular organelle activity difficult by such means. Consequently, these analyses employed ligands and antibodies to identify various intercellular structures with which the SVLP associated.

#### (i) Early endosomes

**Figure 5:**
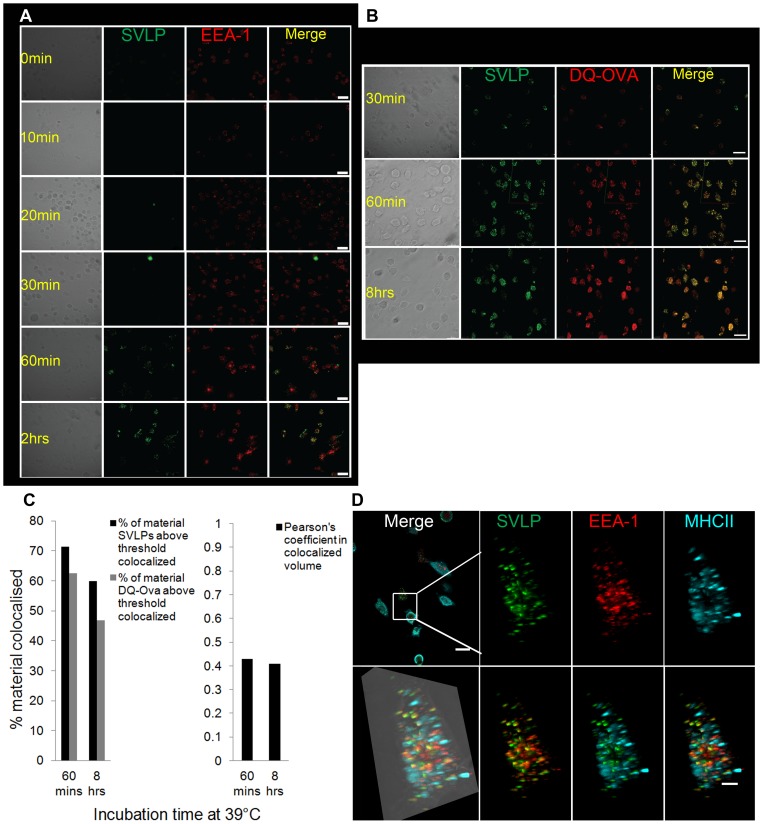
SVLP associating with early endosomes, MHCII and DQ-Ova. (A) Application of quenched SVLP signal to identify endosomal enzyme activity leading to de-quenching of the signal. DC were incubated with 2.5 µg/ml quenched SVLP (green) for different incubation times at 39°C. Samples were fixed, permeabilised and labelled with antibody targeting EEA-1 (red). Acquisition of the images was by confocal microscopy, followed by analysis and merging using IMARIS. Scale bars: 30 µm. (B) Cells were treated as for (A), but also with DQ- Ovalbumin. Acquisition of the images was by confocal microscopy, followed by analysis and merging using IMARIS. Scale bar: 20 µm. (C) Algorithmic co-localisation analysis of SVLP and DQ-Ova performed using IMARIS. (D) Cells were treated as for (A), but stained also for MHCII molecules (blue). Acquisition of the images was by confocal microscopy; high-resolution stacks were prepared using IMARIS. Scale bars: 20 µm (top left), 5 µm (zooms).

EEA-1 positive endosomal structures interacted closely with vesicles carrying SVLP, already clearly visible by 10 min (Fig. 4C). The 3-dimensional images showed EEA-1^+^ structures apparently “engulfing” the SVLP-containing vesicles (Fig. 4D). Interaction with early endosomes was confirmed using SVLP over-conjugated with Alexa_488_ to give a self-quenching of the fluorochrome and absence of signal. Once enzymatic degradation of these SVLP occurred, as in acidifying compartments containing the appropriate cathepsins, the fluorochrome signal should become detectable. A time-dependent appearance of the signal was indeed noted (Fig. 5A). Interestingly, no signal was detectable during first 30 min of incubation at 39°C (Fig. 5A), despite the appearance of unquenched SVLP associating the EEA-1^+^ structures within 10 min (Fig. 4C). By 1 hr, a signal was now consistently apparent from quenched SVLP, seen in what appeared to be vesicular structures. The latter were primarily peripheral within the cell, and often associated with EEA-1^+^ structures, becoming more frequent at 2 hrs (Fig. 5A).

This enzymatic degradation of SVLP in vesicular structures (Fig. 5A) was relatively slow compared with the rapid internalisation (see Fig. 2D). Accordingly, the SVLP accumulation was further analysed together with endocytosed DQ-Ova, another fluorochrome-quenched molecule used to identify the action of endosomal enzymes. Considering the inefficient binding of ova to DC on ice, only the SVLP were pulsed with DC on ice, followed by washing and addition of the DQ-Ova in the medium at all times of incubation. After 30 min at 39°C, many of the SVLP-positive vesicular structures also contained de-quenched (enzymatically cleaved) DQ-Ova (Fig. 5B). The association was particularly strong at 60 min, and continued to be observable at 8 hrs of incubation (Fig. 5B). Application of co-localisation algorithm confirmed this close association (Fig. 5C), giving a Pearson's coefficient in co-localised volume of 0.4. Intriguingly, this value seen at 60 min was retained after 8 hrs incubation at 39°C.

With SVLP being internalised into similar structures as DQ-Ova, suggested similar routes of uptake and processing. The relatively slow rate of the process suggested a role for macropinocytosis; caveolar uptake was unlikely due to the aforementioned absence of SVLP co-localisation with caveolin. Accordingly, the association of the SVLP with other intracellular structures involved in different antigen processing routes was investigated.

#### (ii) MHCII and Microtubules

As in Fig. 5A, de-quenched SVLP was associated with EEA-1^+^ early endosomes by 2 hrs at 39°C (Fig. 5D, red on green signal). In contrast, it was rare to visualise de-quenched SVLP associated with MHCII-rich compartments (Fig. 5D, blue on green signal). This may have reflected an association with MHCII-positive structures typifying more advanced structures in antigen processing, wherein the SVLP had been degraded beyond detectability. Alternatively, a slow rate of processing may have led SVLP into MHCII^+^ compartments at levels too low to be detected.

**Figure 6:**
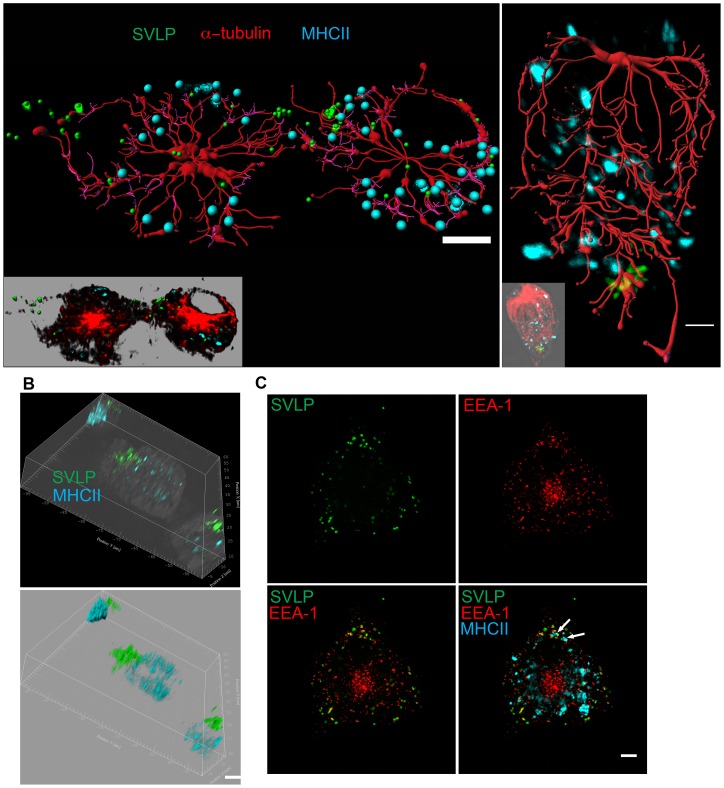
SVLP association with DC microtubules, leading edge and MHCII. (A) Pre-chilled DC were incubated with 2.5 µg/ml SVLP for 20 min on ice, followed by shifting to 39°C for 10 min. Samples were then fixed, permeabilised and labelled with antibody targeting α-tubulin (red) and MHCII (blue). Acquisition was by confocal microscopy; high-resolution stacks were prepared and analysed using Filament Tracer and Spots modules in IMARIS. Visualisation was enhanced using filament tracer (microtubules) and spot (SVLP, MHCII) modules. Scale bars: 5 µm (left), 2 µm (right). (B) Pre-chilled DC were incubated with 2.5 µg/ml SVLP for 30 min on ice, followed by washing and shifting to 39°C for 20 min. Cells were then fixed, permeabilised and labelled with antibody targeting MHCII (blue). Acquisition was by confocal microscopy; high-resolution stacks were prepared using IMARIS. Scale bar: 5 µm. (C) The same cultures were used as in (B), but incubated for 2 hours. Cells were then fixed, permeabilised and labelled with antibody targeting EEA-1 (red) or MHCII (blue). The arrows indicate sites of potential SVLP and MHCII association. Acquisition was by confocal microscopy; high-resolution stacks were prepared using IMARIS. The arrow shows a vesicle in which SVLP and MHCII were present. Scale bar: 5 µm.

DC which had endocytosed SVLP were also stained for MHCII and microtubules. Fig. 6A shows an apposition of MHCII^+^ structures (blue) with microtubules (red), but this was distinct from the SVLP-containing vesicles (green). The majority of the SVLP-containing vesicles were observed appositional to the microtubules (Fig. 6A). It was considered that those SVLP-containing vesicles not associated with microtubules may have been in an earlier stage of internalisation. Certainly, when the SVLP were detectably polarised (as observed with lipid raft association using CTB – see Fig. 3), there was a tight apposition with the microtubules nearer the leading edge of the cell (Fig. 6A, right-hand image).

Further analyses looked at the relative positioning of the SVLP signal in the DC. During the initial 20 min after shifting to 39°C, SVLP were often found internalised at the leading edge of the cell (see Fig. 3 and 6A). Such internalisation yielded a polarised positioning (Fig. 6B), distinctive from the less peripheral and more perinuclear location of the MHC^+^ structures (Fig. 6B). At later time points, such as the 2h shown in Fig. 6C, the SVLP-containing structures, including those associated with EEA-1^+^ structures, were only rarely associating with MHCII^+^ structures. By employing quenched SVLP, so that the signal would only appear upon enzymatic digestion, association of the SVLP signal with MHCII^+^ structures remained rare, in contrast to association with EEA-1^+^ structures (Fig. 6C; see also Fig. 5D). When the occasional interaction with MHC II^+^ structures could be noted (Fig. 6C white arrow), the signal was in the more peripheral “EEA-1^+^ region”, which may explain the more common association with the EEA-1^+^ structures compared with MHCII.

#### (iii) Lysosomes and Endoplasmic Reticulum

Although the results indicate that processing of the SVLP was occurring, albeit at a slow rate, attempts to show association of the SVLP with more degradative lysotracker^+^ organelles was unsuccessful (data not shown). However, DC are reported to express low lysosomal proteolytic activity [9], [24], [25]. As an alternative to loss of detectability during SVLP processing, the SVLP may have been diverted prior to entering lysotracker^+^ organelles. An alternative processing route to maturing endosomes and lysosomes is the transfer into the ER. However, no SVLP signal could be found associated with the ER using ER-tracker or the chaperones calnexin and calreticulin (data not shown).

### Association of SVLP with markers of different endocytic pathways

#### (i) Clathrin

**Figure 7:**
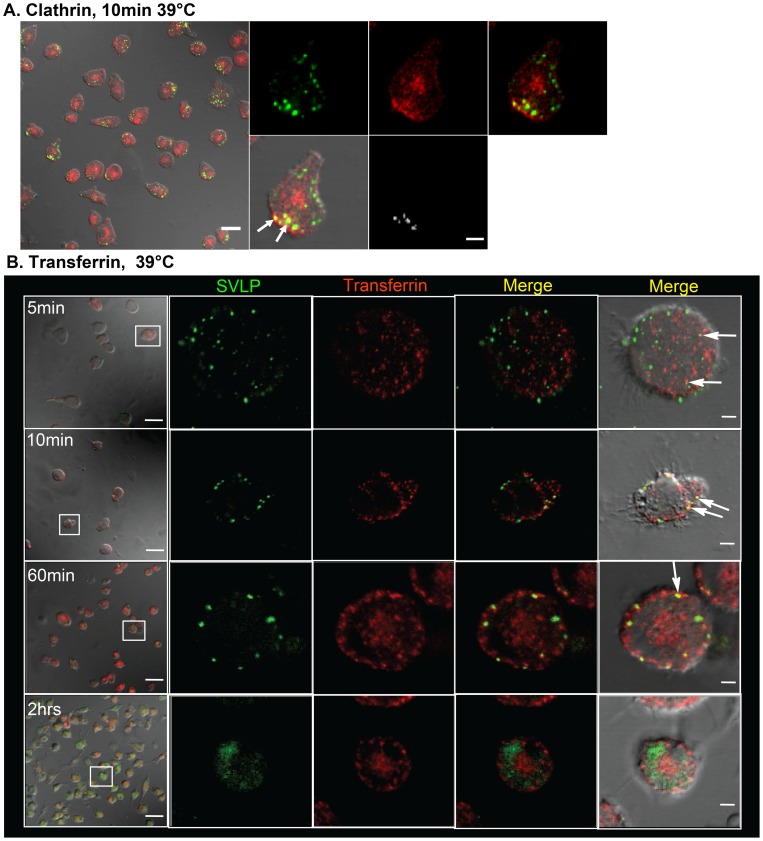
SVLP association with clathrin and transferrin. (A) DC were incubated with 2.5 µg/ml SVLP (green) on ice, shifted to 39°C for different times, fixed/permeabilised, and labelled using antibody targeting clathrin (red). Acquisition was by confocal microscopy; high-resolution stacks were prepared using IMARIS, including algorithmic co-localisation analysis for co-localised voxels. Arrows show co-localisation on the merged image. Scale bars: 20 µm (top-left), 5 µm (zooms). (B) DC were incubated with 2.5 µg/ml SVLP (green) and 10 µg/ml transferrin-546 (red) for 20 min on ice, washed and shifted to 39°C with addition of fresh transferrin-546 for different times at 39°C. Acquisition was by confocal microscopy; high-resolution stacks were prepared using IMARIS. Arrows show co-localisation on the merged image. Scale bars: 30 µm (left-panel) and 5 µm (zooms).

Considering that DC possess different endocytic routes, a role for clathrin-dependent or -independent endocytosis was analysed. Labelling of the DC with antibody against clathrin at different times after the shift to 39°C revealed that the SVLP were mostly associated with structures free of clathrin (data not shown). Only at early time points such as 10 min was there evidence of co-localisation (Fig. 7A). Again, the majority of SVLP-structures were not clathrin^+^, and no such association was observed after this 10 min time point (data not shown).

#### (ii) Transferrin

Alexa-labelled transferrin was used as a marker for clathrin-mediated endocytosis, as well as an aid for identifying sorting endosomal structures; following the shift from ice to 39°C, unbound SVLP were removed, but presence of the transferrin was maintained for the different time points of incubation. The earliest time point of 5 seconds (plus the time for washing) after the shift to 39°C showed no association of transferrin and SVLP (data not shown). From 5 min, both transferrin and the SVLP were becoming less associated with the cell periphery, but there were still no signs of co-localisation (Fig. 7B, top row). Between 10 and 60 min, the transferrin continued to be translocated from the periphery (replaced presumably by newly endocytosed transferrin at the periphery), but the SVLP tended to remain more peripheral than the transferrin (Fig. 7B, 2^nd^ and 3^rd^ rows). It was during this period that SVLP associating with transferrin-containing vesicles was noted, also more peripheral (Fig. 7B, arrows). By the 2 hrs time point, the SVLP-containing structures were showing evidence that they were now translocating from the cell surface in larger numbers, but were distinct from the transferrin-bearing structures (Fig. 7B, 4^th^ row), Interestingly, a number of particularly perinuclear transferrin^+^ structures were seen, but the SVLP remained to the peripheral side of these (Fig. 7B, 4^th^ row).

#### (iii) Dextran

**Figure 8:**
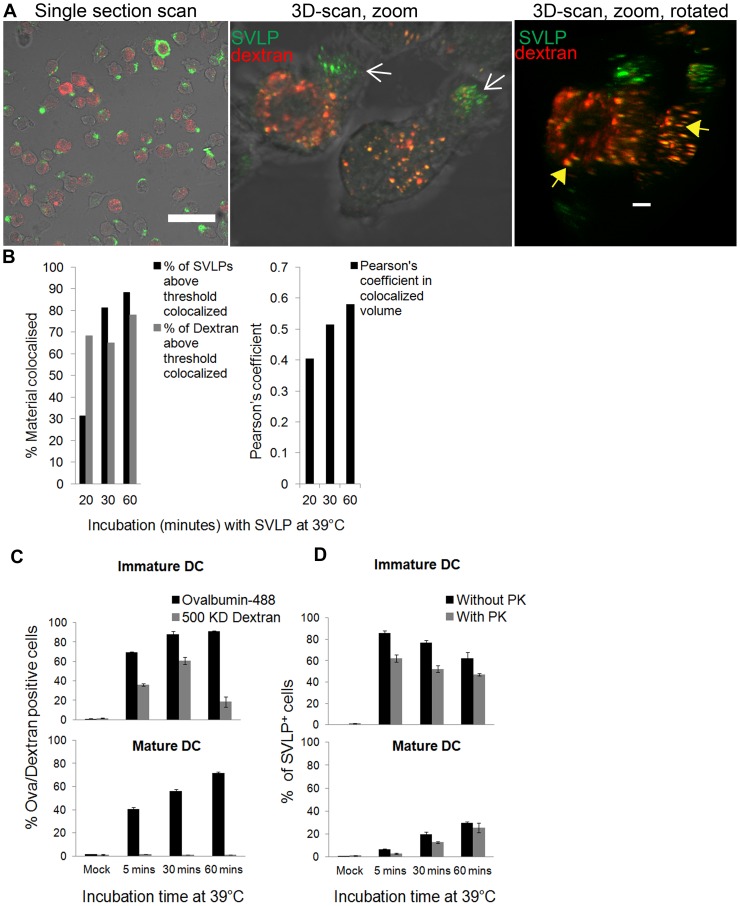
SVLP association with high molecular weight dextran and its uptake by mature or immature DC. (A) DC were incubated with 50 µg/ml high molecular weight dextran for 30 min at 39°C, followed by “on ice” for 30 min. Cells were then treated with 2.5 µg/ml SVLP and incubated for 30 min, then shifted to 39°C for different times. Acquisition was by confocal microscopy; high-resolution stacks were prepared using IMARIS. White arrows: SVLP near the leading edge. Yellow arrows: Dextran^+^ vesicles in apposition with SVLP-vesicles. Scale bars: 50 µm (top-left), 3 µm (zooms). (B) Algorithms of SVLP co-localisation with dextran. (C) Four-day old DC were treated or not with interferon-α (1000 U/ml) and lipopolysaccharide (10 µg/ml) for 24 hrs to mature. High molecular weight dextran (50 µg/ml) or ovalbumin (10 µg/ml) was added, and incubated for 5, 30 or 60 min at 39°C. The number of positive cells was measured by flow cytometry; each bar represents means of three values and standard deviation. (D) Internalisation of SVLP (1 µg/ml) by the immature and mature DC used in (C). SVLP were added for 30 min on ice, then shifted to 39°C for different times, when the cultures were chilled and treated or not for 30 min with PK on ice. The number of positive cells was measured by flow cytometry; each bar represents means of three values and standard deviation.

The evidence pointing to the clathrin-independent endocytic process of macropinocytosis was interesting for SVLP considering that many viral pathogens employ this route to infect host cells including DC [26]. Accordingly, high molecular weight dextran was employed as a marker for macropinocytosis [27], due to its accumulation in macropinosomes; the high molecular weight of the dextran prevents its translocation from the vesicular structures. Cells were given high molecular weight dextran 30 min at 39°C prior to adding the SVLP, allowing the dextran to accumulate, and ensure that there would not be confusion with any dextran still entering the cells. DC were then washed and pulsed with SVLP on ice for 20 min, unbound SVLP washed away, and the cells shifted to 39°C for different time points. By 20 min of incubation at 39°C most of the SVLP^+^ vesicles were associating with dextran^+^ vesicles (Fig. 8A, yellow dots). Interestingly, the SVLP^+^ vesicles still at the leading edge of the cells were distinct from the dextran-containing vesicles (Fig. 8A, white arrow). It was also observed in 3D-analyses that some dextran^+^ vesicles were seen in apposition with SVLP-vesicles (Fig. 8A, right-hand image, yellow arrows). Nevertheless, algorithmic analysis of co-localised voxels demonstrated a high confidence in co-localisation (Fig. 8B), and this was time-dependent.

### SVLP internalisation by immature and mature DC

Further analysis of macropinocytosis used the characteristic of mature DC having reduced activity compared with immature DC [28]. The uptake of SVLP by mature and immature DC was therefore compared, using DC matured with interferon-α (1000 U/ml) plus lipopolysaccharide (10 µg/ml) for 24 hrs: the immature DC were left untreated. For macropinocytosis, the uptake of high molecular weight dextran (500 KD) and ovalbumin were used as markers. Mature DC did not endocytose high molecular weight dextran, unlike immature DC, confirming the relative states of maturation (Fig. 8C). Ovalbumin uptake was partially reduced in mature DC compared to immature DC (Fig. 8C), reflecting its known uptake by macropinocytosis plus other endocytic routes. Internalisation of SVLP (to become PK-resistant) by mature DC was also significantly reduced, whereas immature DC were 60% positive for SVLP within 5 min (Fig. 8D). Although these results indicate a major role for macropinocytosis in DC internalisation of SVLP, this is not the only endocytic process involved. Mature DC did internalise SVLP, but to a lower level and with a much slower kinetics.

## Discussion

DC express a wide variety of receptors to constitutively sample their environment, and employ different endocytic pathways to endocytose antigens. In contrast to more degradative cells, such as macrophages, DC display a lower lysosomal proteolytic activity and show greater control of proteasees process [9], [24], [25]. Indeed, Delamarre et al related this limited protease activity of DC to their slower in vivo degradation of internalized antigens and retention of antigen in lymphoid organs for extended periods. They remarked that this limited lysosomal proteolytic activity would favour antigen presentation, and help explain the DC ability for efficient accumulation, processing, and dissemination of antigens.

Considering these characteristics of DC, we sought to determine how DC would process a complex antigen resembling a small synthetic virus-like particle – SVLP. Many current vaccines act against viruses, and many vaccines are based on virus particles. SVLP have a high immunogenicity in mice, rabbits [17] and pigs (R.S., unpublished data); of particular note is that unlike most non-replicating vaccines, the SVLP do not require formulation with adjuvant. It was considered that analysis of how DC handle a more complex virus-like structure would be informative; by using SVLP, the analyses of DC function would not be complicated by any adjuvant-formulation effect.

SVLP were bound rapidly by DC, even on ice, more efficiently than for high molecular dextran, ovalbumin or transferrin. Internalisation of the SVLP was initially determined in terms of PK-resistance (internalised). Although the PK did not remove the DC receptors binding SVLP (whereas pronase did), the PK did efficiently destroy any SVLP still bound to the DC surface. Following binding to the DC on ice, then washing to remove unbound SVLP, further incubation at 39°C showed that the SVLP gradually became resistant to the PK-treatment, but in contrast with the rapid binding, internalisation was more gradual. This was reflected by cell fractionation analyses. Association with the cytoskeletal fraction was strongest at earlier time points, whereas association with the cytoplasmic fraction was not noted until after 30 min. Membrane association was observed throughout the 4 hrs observation period, probably reflecting the initial interaction with the plasma membrane, followed by vesicular membranes relating to internalisation. The SVLP signal faded at later time points, but the loss of signal was still gradual over a number of days. This kinetics of uptake and probable processing is consistent with the report that DC process antigen slowly [9]. However, it was still unclear if the slow kinetics were related to intracellular processing or simply the rate of internalisation.

Primary DC and monocyte-derived DC are inefficient at functional DNA transfection [23], possibly due to the impermeability and resistance of their nuclear membranes. It was considered inappropriate to employ cell lines, because the aim of the work was to characterize how primary DC handled a complex antigen with proven in vivo efficacy. Accordingly, the intracellular compartments with which the SVLP associated were analysed using ligands and antibodies for their identification.

SVLP interacted with lipid rafts on the DC surface. This was concluded due to their patchy association with the cell surface on ice, accumulation at the leading edge of the cell on shifting to 39°C, and co-localisation with CTB, which is known to interact with lipid rafts [21]. Co-localisation of the SVLP with the CD9 marker associated with lipid rafts of human monocytes [22], and sensitivity to MBCD treatment, known to interfere with cholesterol restoration, confirmed that SVLP endocytosis by DC was dominated by lipid raft-dependent processes. These were caveolin-independent, because there was no co-localisation of SVLP-containing structures with caveolin-1 (data not shown). Following internalisation, there was an association with EEA-1^+^ structures, which would relate to macropinosomes interacting with early endosomes initiating antigen processing [5], [6], [7], [8]. The association of the internalised SVLP with EEA-1^+^ structures was a gradual process, the frequency increasing as time progressed. Moreover, not all SVLP-containing structures co-localised with EEA-1^+^ structures, even at later time points. The nature of this EEA-1 association was studied further using SVLP in which the fluorochrome signal was self-quenched, and therefore required degradation for de-quenching. When the processed SVLP was de-quenched, the signal did co-localise with EEA-1, but this only became apparent between 30 and 60 min (depending on the experiment), more frequent by 1 to 2 hrs. This kinetics followed that of DQ-Ova, a self-quenched entity employed for detecting endosomal-processing events, the high degree of overlap indicating similar processing pathways. This confirmed slow enzymatic processing, but also indicated that the SVLP-containing vesicles not associated with EEA-1 had probably not interacted with early endosomes.

In contrast to the strong association with EEA-1^+^ structures, even at later time points it was difficult to visualise association with MHCII^+^ structures. Yet, the SVLP signal was aligning with microtubules, confirming that intracellular transport was probable. Nevertheless, the SVLP-containing vesicles, even when EEA-1^+^, tended to remain more peripheral rather than trafficking towards a more perinuclear region of the cell. The occasional SVLP-MHC interaction that was noted, tended to be more at the peripheral edge of region containing MHCII^+^ structures (intermediate between the peripheral area occupied by most of the SVLP and a more perinuclear region, also EEA-1^+^). With the peripheral SVLP signal not declining rapidly, the endocytic process was apparently maintaining the SVLP-containing structures in this region, even after interaction with EEA-1^+^ vesicles.

Being indicative of macropinocytosis, with the advancement of processing appearing gradual, this could explain why the observed interaction with the MHCII^+^ structures was a rare event. Visualisation of further processing may have proven difficult due to a slow processing, when the amount of SVLP moving forward in the processing chain was low, perhaps often too low to be visualised. The same argument may be true for association with the ER. This can also occur by translocation for macropinosomes [4], before the acidification and proteolytic processing is too far advanced. Although it was difficult to observe an association of SVLP with the ER, there may have been a low level of translocation, the number of events or amount of material involved being too low for detection.

Although the results were indicating an important role for lipid-raft mediated macropinocytosis, there was evidence of an early involvement for a clathrin-dependent route. Nevertheless, most SVLP were not associated with clathrin^+^ structures, and the latter were only visible during the first 10 min. This implies that clathrin-dependent endocytosis is of minor relevance to the processing of SVLP for antigen presentation; indeed, the early clathrin-dependent uptake may be leading the SVLP into a rapid degradative process, rather than the slower and more progressive antigen-processing route. The results with transferrin would also argue in this direction. While transferrin was continually observed translocating from the periphery, the SVLP tended to remain more peripheral than the transferrin. Some peripheral vesicles did contain both SVLP and transferrin, but this was rather late – at the 60-min time point. This may reflect diversion of some SVLP-containing vesicles into sorting endosomes. Although such structures were in the minority, such a possibility relates to the sorting of antigen for delivery to B-lymphocytes [1], [29].

The importance of macropinocytosis in SVLP internalisation by DC was confirmed using high molecular weight dextran [27], which should remain blocked within macropinosomes. Allowing dextran to accumulate in macropinosomes, SVLP-containing vesicles entering at the leading edge of the DC were not associated with dextran, confirming their early endocytic stage. By 20 min at 39°C, most of the SVLP co-localised with the dextran^+^ vesicles, strongly indicative of SVLP accumulation in macropinosomes. This would certainly relate to the importance of macropinocytosis for the entry of viruses into DC [26]. Use of maturing DC for their reduced macropinocytic activity [28] further confirmed the importance of macropinocytosis during SVLP uptake. Mature DC were inferior to immature DC for endocytosis of SVLP; in contrast to high molecular weight dextran, the uptake of which was abrogated in mature DC, there was a residual uptake of SVLP. This internalisation by mature DC was much slower than with immature cells, and never reached the levels obtained with the latter. It has been reported that mature DC continue to employ receptor-mediated endocytosis upon maturation [30], despite macropinocytosis being significantly reduced. Such observations demonstrate that macropinocytosis may be the major route for endocytosis of SVLP by DC, but other endocytic processes, albeit less efficient, can be involved. Regardless, internalisation of SVLP by DC is a gradual process, typical of what has been reported for DC antigen processing [9].

The present work sought to characterize how DC interact with a complex, virus-like antigenic structure – SVLP – which is an efficacious immunogen requiring no adjuvant to induce adaptive immunity in mice, rabbits [17] and pigs (R.S., unpublished data). It was considered that this interaction would highlight the elements contributing to efficient handling by DC leading to the observed in vivo efficacy. Overall, DC interacted rapidly with the SVLP, but displayed a more gradual kinetics for uptake and processing, the latter relating to reported in vivo characteristics for DC [9]. Endocytosis was a relatively slow process dominated by caveolin-independent, lipid raft polarisation, leading the SVLP into a peripheral location. The processing was dominated by macropinocytosis, although non-macropinocytic events were likely in the background. The processing was characterized by slow kinetics, involving fusion with EEA-1^+^ early endosomes in the periphery, and followed a common path to that with dextran. This did not lead into the more degradative pathway employed with transferrin, nor was there any observable association with lysosomes. The rare localisation with MHCII-structures and the ER may reflect a loss of signal once the material reached these organelles, due to proteolytic cleavage. It is also possible that such rare localisation reflected a slow progress of material into these structures, at a level too low to be detected. The prolonged association with peripheral structures, which were not all EEA-1^+^, and the slow processing relate to macropinosomal retention of antigen for slow antigen processing [9], and would allow for entry into sorting compartments favouring transfer of antigen to B lymphocytes [1], [29]. Certainly, the processing was effective, as witnessed by the immune responses induced by SVLP in vivo. These characteristics of how DC handle a complex virus-like structure such as SVLP do relate to efficacious processing, and the characteristics DC require for efficient induction of immune responses.

## Materials and Methods

### Ethics Statement

Blood donor pigs were used for the supply of blood, from which blood cells were prepared, and used for the generation of monocyte-derived DC. This operated under permission from the Canton of Bern, Switzerland through the animal licence 112/09.

### Porcine monocyte-derived DC

Porcine monocyte-derived DC were prepared as previously described [31], from specific pathogen-free pigs held at the Institute's facility. Briefly, these were prepared from peripheral blood cells using CD172a-magnetic sorting to isolate monocytes, then culturing for 3–5 days with recombinant porcine GM_CSF and IL-4 as described [32]. Monocytes are characterised by high expression of CD172a (Fig. S1C), and the sorting yields >90% purity. Upon culture in GM-CSF and IL-4, we obtained ≥95% DC purity, characterized as described [32], [31]. Cell culture medium and serum was obtained from Invitrogen (Basel, Switzerland).

### SVLP

Lipopeptide buildings blocks were prepared as described previously [17], Briefly, the peptide GGIEKKIEAIEKKIEAIEKKIEAIEKKIEAIEKKIAKMEKASSVFNVVNSKKKC-^D^A-amide (0.25 mmol scale) was assembled on an ABI 433A peptide synthesizer using standard Fmoc chemistry and Rink amide MBHA resin pre-loaded with Fmoc-D-Ala (substitution: 0.3 mmol/g). After assembly and removal of the N-terminal Fmoc-protecting group using 20% piperidine in DMF, the lipid 1,3-dipalmitoyl-glycero-2-phosphoethanolamine-N-succinic acid [17] was coupled manually using PyBOP/HOBt/DIEA for activation. The resin was then washed with DMF (3x), CH_2_Cl_2_ (3x) and CH_3_OH (3x), dried in vacuo over KOH pellets and treated with CF_3_COOH/thioanisole/ethanedithiol/H_2_O/i-Pr_3_SiH (75∶10∶10∶4∶1, 10 ml). The lipopeptide was precipitated with *i*Pr_2_O (cooled to −20°), washed 3x with *i*Pr_2_O, dried and purified by reverse phase HPLC using an UP10WC4/25M preparative C4 column (Interchrom, 250×21.2 mm, 5 µm particle size, 300 Å pore size). For cell-uptake and intracellular trafficking studies, SVLP were labelled with Alexa-Fluor 488 C_5_ maleimide (Life Technologies, Zug, Switzerland). Remaining free cysteines were blocked with N-ethylmaleimide (Sigma). For some experiments, in order to observe degradation in acidifying compartments, over-labelled SVLP were prepared by conjugating excess Alexa-Fluor 488 C_5_ maleimide. The molecular weights of the lipopeptide and Alexafluor 488 conjugate were confirmed by mass spectrometry (MALDI-TOF and/or ESI-MS) and the purity was >96% by analytical reversed phase HPLC. The size distribution of SVLP was monomodal with narrow size-dispersion and a hydrodynamic radius [14] of 11.2–12.8 nm by dynamic light scattering (DLS). Unless stated otherwise, the SVLP were used at 1 µg/ml for Flow Cytometry and 2.5 µg/ml for confocal microscopy.

### Proteinase K and pronase treatment of DC

In order to quantify SVLP internalisation by DC, flow cytometry was employed. To this end, it was important to differentiate between the surface bound and internalised SVLP. Accordingly, Proteinase K (PK) obtained from Sigma was employed. PK was titrated with DC on ice, to determine the least toxic concentration, PK induced toxicity being observed by PI staining. Least toxicity was seen with 0.2 mg/ml of PK. Therefore, 0.2 mg/ml of PK was used in all the experiments. PK was always used under cold conditions on ice in order to minimise cell damage. Pre-chilled DC were incubated with SVLP on ice for 20 or 30 min, or without SVLP were washed with 2 ml ice cold phosphate-buffered saline (PBS). Cell pellet was re-suspended in 500 µl of cold Dulbecco's MEM (DMEM) and incubated on ice for 20 min. Then PK (0.2 mg/ml) was added followed by 30 min incubation on ice. Then, 10% (v/v) porcine serum (PS) was added in order to inactivate PK activity. Cells were then washed with 2 ml ice cold cell wash or PBS by centrifuging at 350× g. Cells were re-suspended in cell wash to analyse by flow cytometry or in DMEM to incubate with SVLP.

DC were also treated with pronase (Roche), for stripping protein receptors. DC (0.5×10^6^/ml) were treated with different concentrations of pronase and incubated at 39°C for 25 min, followed by 5 times washing with 2 ml ice cold cell wash containing 10% (v/v) PS. DC were immediately re-suspended in 500 µl cold DMEM and shifted on ice for 30 min, followed by addition of SVLP or CTB and incubated for 20 min on ice. Cells were then fixed with 4% (w/v) PFA, or surface labelled with antibody against CD172a, CD14 or MHCI; replicates of cells stained with the former two antibodies were treated with 3mM NaN_3_ to impair receptor recycling.

### Antibodies and markers

Considering the difficulties in expressing tagged proteins in primary DC using transfected DNA plasmids, visualisation of different cell organelles had to rely on direct labelling in situ. Accordingly, labelling of different intra-cellular organelles employed the following reagents: Major Histocompatability Complex II (MHCII) containing vesicles were labelled with antibody against MHCII molecules (mouse monoclonal 1F12 IgG2b; BD Transduction Laboratories), microtubules were labelled with anti α-tubulin (mouse monoclonal IgM; Santa Cruz Biotechnology); early endosomes were labelled with antibody against Early Endosome Antigen 1 (EEA-1) (Mouse Anti-EEA-1; BD Transduction Laboratories); clathrin coated pits were labelled with anti-clathrin antibody (mouse anti-clathrin Heavy Chain; BD Transduction Laboratories); Endoplasmic Reticulum (ER) was labelled with antibody against Calnexin (pAB Rabbit; Enzo Life Sciences); lipid rafts were labelled with antibody against CD9 (Serotech) or cholera toxin B (CTB)-Alexa_546_ (Life Technologies, Zug, Switzerland); CTB was used at a final concentration of 5 µg/ml. Isotypespecific conjugates labelled with Alexa-fluro dye were used from Life Technologies. Markers used for defining different endocytic pathways included biotinylated high molecular weight dextran-500 KDa (Sigma) for macropinocytosis, transferrin from human serum labelled with Alexa_546_ for clathrin-mediated endocytosis and recycling endosomes, CTB-Alexa_546_ for marking lipid rafts, DQ-Ovalbumin (DQ-Ova) to mark acidifying compartments, and ovalbumin labelled with Alexa_488_ for multiple endocytic routes; all from Life Technologies.

### Immunolabelling

Four day-old DC (0.2 million cells/well) were seeded in 8-well fibronectin-coated Labteks (Nunc, Wiesbaden, Germany) with 200 µl of DMEM per well. For labelling of intracellular organelles, DC were fixed with 4% (w/v) paraformaldehyde (PFA) and washed twice with 0.1% (w/v) saponin, followed by incubation with the primary antibody (see above) diluted in 0.3% (w/v) saponin for 30 min on ice. Cells were then washed twice with 0.1% (w/v) saponin, followed by addition of an Alexa fluor-labelled conjugated secondary antibody diluted in 0.3% (w/v) saponin, with 30 min incubation on ice. Cells were then washed twice with 0.1% (w/v) saponin in cell wash. DC were then mounted in Mowiol and analysed by confocal microscopy.

The exception to the above routine was the procedure to label microtubules. Cells were washed twice with microtubule stabilising buffer (MSB), and then permeabilised with 1% (w/v) Triton X-100 in MSB for 1 min at room temperature. The cells were then washed and stained with the antibody against α-tubulin in cell wash for 30 min on ice, washed twice, and then given the Alexa fluor-labelled conjugated secondary antibody in cell wash for another 30 min on ice. After final washing, the cells were mounted in Mowiol and analysed by confocal microscopy.

### Confocal Microscopy

Confocal microscopy employed a Leica TCS SL microscope and LCS software (Leica Microsystems AG, Glattbrugg, Switzerland). All images were acquired using a 63× oil-immersion objective; analysis used IMARIS-7.4.2 software (Bitplane AG). For co-localisation analysis, high-resolution images acquired at optimum voxel size were used, and automatic threshold applied. Percentage material co-localised and Pearson's coefficient were also calculated. All microscopy analyses employed threshold subtraction and gamma-correction, relating to the negative controls that no false positive emissions were present. Some images had analyses with filament tracer and spot modules of IMARIS, to enhance their visualisation.

### DC Maturation

Four day-old DC were treated with interferon-α (1000 U/ml) (in house) and lipopolysaccharide (LPS) (10 µg/ml) (Sigma) for 24 hrs at 39°C. Cells were then harvested and pulsed with SVLP on ice for 20 min., followed by washing and incubation at 39°C for different time points. Cells were then treated with PK or were directly analysed by flow cytometry.

### High molecular weight dextran and CTB-Alexa_546_ markers

Cells were incubated with biotinylated high molecular weight dextran (50 µg/ml) for 30 min at 39°C, followed by washing and incubating on ice for 30 min. SVLP (2.5 µg/ml) were then added and further incubated on ice for 20 min. Cells were then washed and shifted to 39°C for different time points. DC were fixed with 4% (w/v) PFA at different times. Cells were then permeabilised (as above) and labelled with streptavidin-546 (Invitrogen) diluted in 0.3% (w/v) saponin.

Four-day old DC were pre-incubated on ice for 30 min, followed by addition of SVLP, or CTB-Alexa_546_, or anti-CD9 antibody, or all together. The cells were then further incubated on ice for 30 min and then washed twice with cold PBS. Warm DMEM was then added and cells were shifted to 39°C for different incubation times, followed by fixing and mounting (as above).

### DC treatment with inhibitors

DC were treated with 20 mM methyl-β cyclodextran (MBCD; Sigma) for 20 min at 39°C. Cells were then shifted on ice and incubated for 30 min. Then fresh medium with 2 mM MBCD was added to prevent cholesterol restoration, followed by addition of SVLP, with incubation for 20 min on ice. Cells were then washed and fixed with 4% (w/v) PFA, or stained labelled streptavidin prior to fixation. One set of samples were treated with PK and the other not.

### Flow Cytometry

FACS Calibur analytical FCM (Becton Dickinson, Basel, Switzerland) was used for acquiring data, and CellQuest Pro software (Becton Dickinson) for analysis. Always 10,000 gated live cells were acquired. Statistical analysis employed the FlowJo software (Treestar, San Carlos, CA), which was also used to prepare the flow cytometry plots.

### Statistical Analysis

For statistical analysis, the Student *t*-test was performed, using SigmaPlot Versin 11.0. Differences were considered to be significant when p≤0.05.

## Supporting Information

Figure S1 **SVLP structure and PBMC sorting.** (A) Structure of SVLP construct. (B) Purity of cell fractions isolated from PBMC by magnetic sorting using CD172a to isolate monocytes.
(TIF)

Figure S2 **Influence of MBCD on SVLP and CTB interaction with DC.** (A) Flow Cytometry histograms showing the uptake of SVLP by DC, triplicate samples, in the absence of MBCD or after pre-treatment with 20 mM MBCD for 20min at 39°C prior to washing and adding the SVLP for 20min on ice. For reference, the cell control is shown as the solid grey histogram. The “Overlay” shows just the results with (coloured) and without (grey/black) MBCD. (B) As in (A), but using CTB in place of SVLP. (C) As in (A), but comparing a particular stock of SVLP used at 1 µg/ml and 10 µg/ml.
(TIF)
