# Supplementary figures and images for: Synthetic Virus-Like Particles Target Dendritic Cell Lipid Rafts for Rapid Endocytosis Primarily but Not Exclusively by Macropinocytosis
Source: PLoS One. 2012 Aug 14;7(8):e43248. doi: 10.1371/journal.pone.0043248 (PMC3419204; doi:10.1371/journal.pone.0043248)

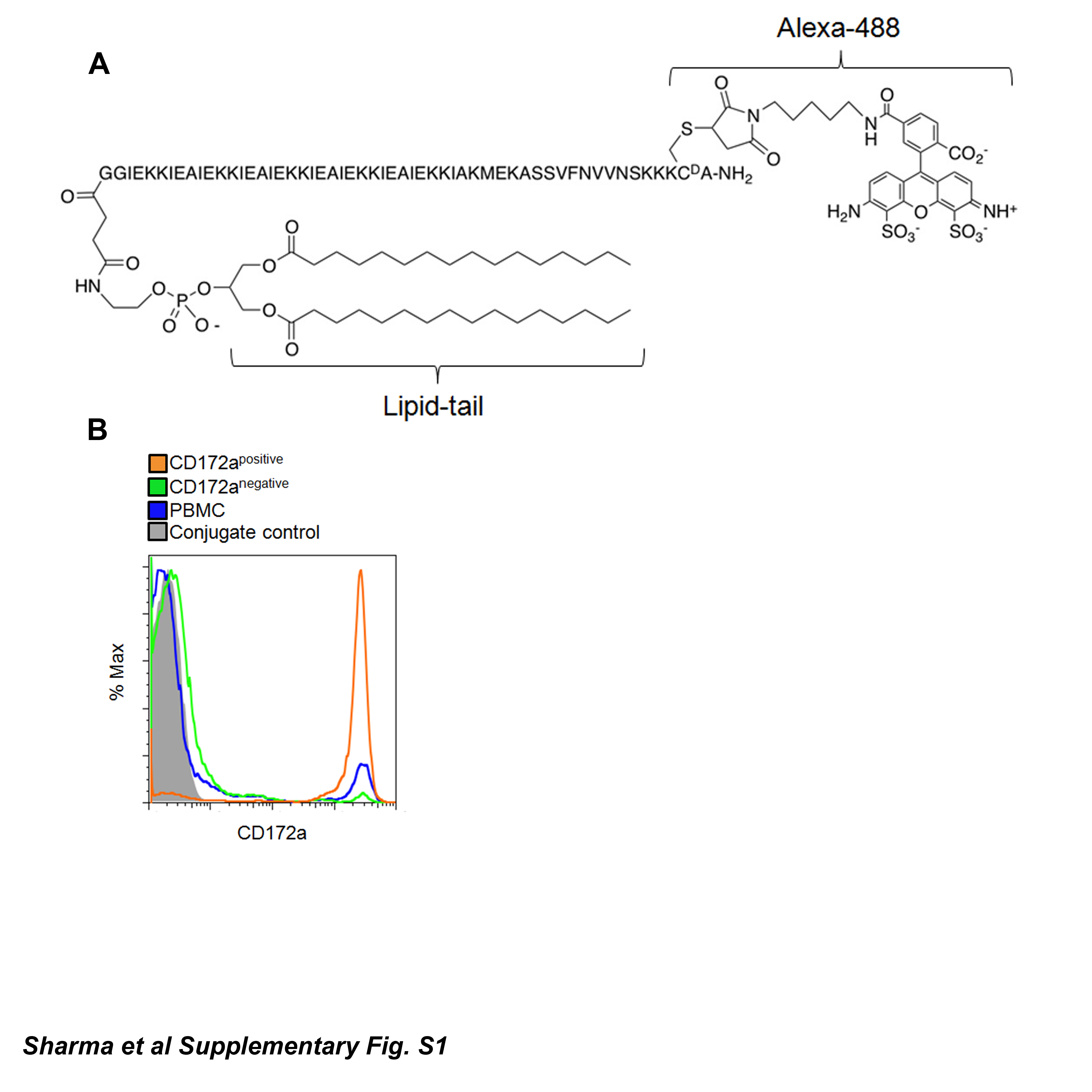

Supplement: Figure S1 — SVLP structure and PBMC sorting. (A) Structure of SVLP construct. (B) Purity of cell fractions isolated from PBMC by magnetic sorting using CD172a to isolate monocytes. (TIF) [file pone.0043248.s001.tif]

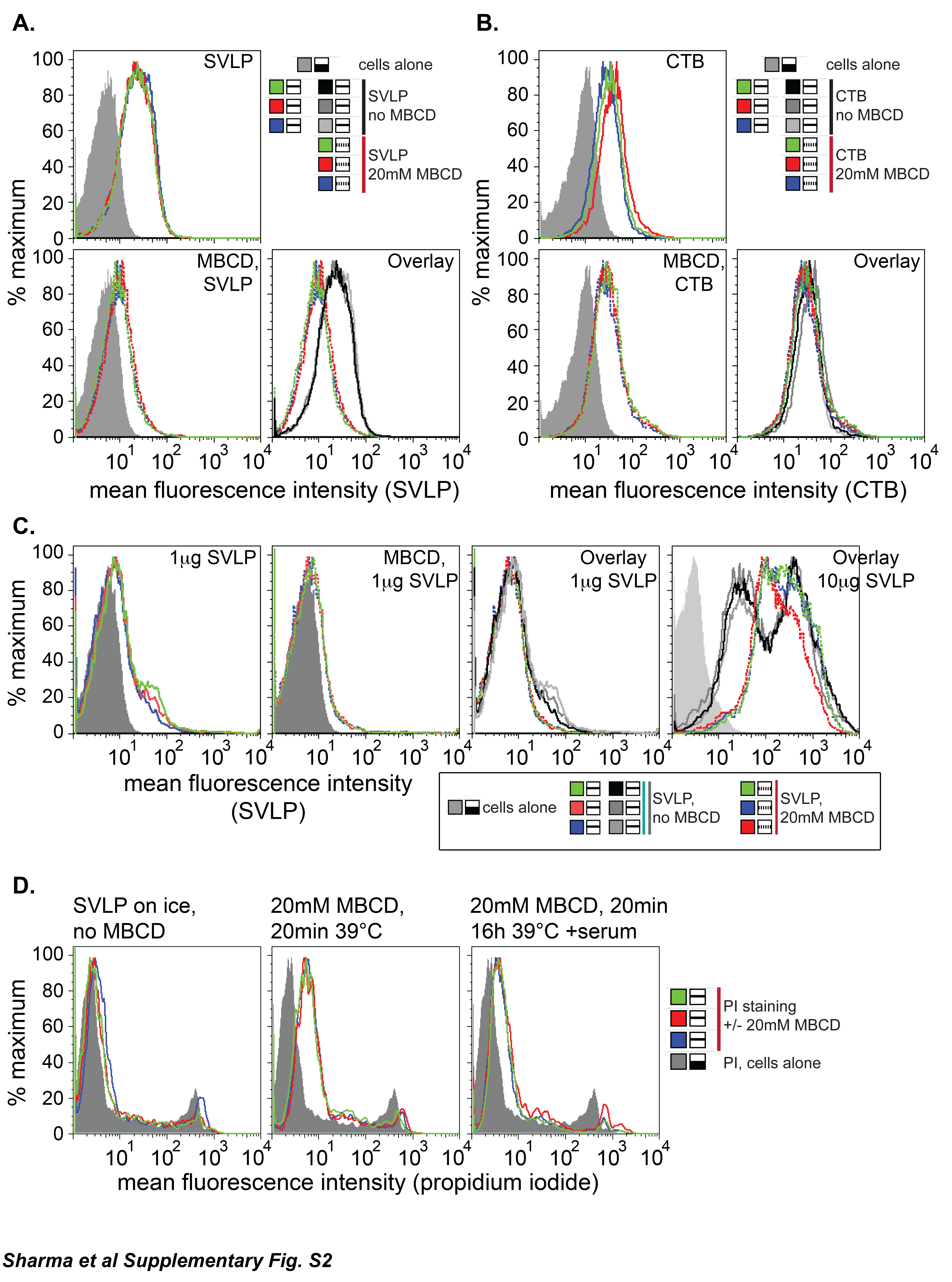

Supplement: Figure S2 — Influence of MBCD on SVLP and CTB interaction with DC. (A) Flow Cytometry histograms showing the uptake of SVLP by DC, triplicate samples, in the absence of MBCD or after pre-treatment with 20 mM MBCD for 20min at 39°C prior to washing and adding the SVLP for 20min on ice. For reference, the cell control is shown as the solid grey histogram. The “Overlay” shows just the results with (coloured) and without (grey/black) MBCD. (B) As in (A), but using CTB in place of SVLP. (C) As in (A), but comparing a particular stock of SVLP used at 1 µg/ml and 10 µg/ml. (TIF) [file pone.0043248.s002.tif]
